# HLA Polymorphism Affects Risk of *de novo* Mutation of *dystrophin* Gene and Clinical Severity of Duchenne Muscular Dystrophy in a Southern Chinese Population

**DOI:** 10.3389/fneur.2018.00970

**Published:** 2018-11-15

**Authors:** Huan Li, Lulu Xiao, Liang Wang, Jinfu Lin, Min Luo, Menglong Chen, Ruojie He, Yuling Zhu, Cheng Zhang

**Affiliations:** ^1^Department of Neurology, National Key Clinical Department and Key Discipline of Neurology, The First Affiliated Hospital, Sun Yat-sen University, Guangzhou, China; ^2^Department of Tissue Typing Center, Nanfang Hospital, Southern Medical University, Guangzhou, China; ^3^Laboratory Medicine Center, Nanfang Hospital, Southern Medical University, Guangzhou, China; ^4^Department of Neurology, The First Affiliated Hospital of Jinan University, Guangzhou, China

**Keywords:** Duchenne muscular dystrophy (DMD), HLA-A^*^29:01, HLA-B^*^07:05, HLA-A^*^02:01, allele frequency, *de novo* mutation, severity

## Abstract

Immune-mediated pathology has been thought to be an important factor contributing to Duchenne muscular dystrophy (DMD). Allele frequencies of certain HLA types are known to differ between patients with dystrophinopathies and healthy controls with low-resolution HLA gene typing data in limit reports. Using Polymerase chain reactionsequence based typing (PCR-SBT) to genotype 64 children with DMD in *HLA-A*, -B,-C, -DRB1, and *-DQB1* locus and 503 healthy controls in *HLA-A*, -*B, -DRB1* locus, this study aimed to investigate associations of specific *HLA* alleles with, and their possible roles in the development and clinical phenotypic severity of DMD. The χ^2^ test was used to evaluate the distribution of allele frequencies in *HLA-A*, -*B, -DRB1* locus between the patients and healthy controls. A significantly higher frequency of HLA-B^*^07:05 was found in children with DMD compared to that in controls (OR = 16.2, 95%CI = 2.9–89.3, *P* < 0.046). More importantly, significantly higher frequencies of HLA-A^*^29:01 (OR = 77.308, 95%CI = 6.794–879.731, *P* < 0.0160) and HLA-B^*^07:05 (OR = 60.240, 95%CI = 9.637–376.535, *P* < 2.41^*^10^−3^) was found in patients with *de novo* mutations (*n* = 14) compared to controls while no difference of HLA alleles frequency ware indicated between patients with inherited mutation and control. The result indicates that *HLA* alleles is associated with pathogenesis of DMD especially DMD with *de novo* mutation. We use Vignos scale to estimate the lower limb motor function of patients. The impact of HLA alleles on score of Vignos scale of DMD children was estimated by multiple linear regression. Our study indicates that HLA-A^*^02:01 may have a dampening effect on the clinical phenotypic severity of DMD, evidenced by the presence of HLA-A^*^02:01 being associated with lower Vignos score. Our study demonstrates that certain *HLA* alleles are indeed associated with the pathogenesis and clinical phenotypic severity of DMD.

## Introduction

Duchenne muscular dystrophy (DMD) is an X-linked genetic disorder caused by mutations in the *dystrophin* gene (*DMD*, MIM# 300377). The condition affects 1 in every 3,600–6,000 live male births, and is characterized by pseudohypertrophy of the gastrocnemius muscle, progressive muscle weakness, and muscular atrophy (1). Patients usually develop motor dysfunctions at the age of 3–5 years, lose the ability to walk before 15 years of age, and die of respiratory or cardiac complications in their twenties or thirties (1). Until now, there is still no curative treatment for DMD though several pharmacological and gene therapy approaches have been tested over the years (2). Only glucocorticoid therapy is proved to be effective to slow down the disease progression though the continued use of the therapy brings many side effects (3).

The *DMD* gene encodes dystrophin, a large (427 kD) cytoskeletal membrane protein that provides structural stability to the plasma membrane of myofibers. The protein is a vital component of the dystrophin glycoprotein complex (DGC) and protects the myofibers from contraction-induced injury (4). A lack of dystrophin making myofibers cannot withstand the stresses of contraction/relaxation cycles; leading to membrane damage, repeated cycles of necrosis and regeneration, and inflammation, that finally lead to the premature death of myofibers and the replacement of muscle tissue by fibro-fatty connective tissue (5). Mechanical injury and membrane defects caused by the lack of dystrophin are the prime causes of DMD symptoms, but they do not fully explain the varied clinical phenotypes of DMD. In human patients with DMD, as well as in animal models of the disease (such as mice, dogs, and cats), there are remarkably diverse variations in the age of onset and severity of the muscle disease (6). Immune-mediated pathology has been thought to be an important factor contributing to DMD. The innate immune system is found to be strongly activated in DMD patients even before the onset of clinical symptoms; this activation includes accumulation of immune cell including CD4^+^ and CD8^+^ T cells, macrophages, eosinophils, and natural killer T cells, altered signaling pathways via Toll-like receptors (TLRs) and nuclear factor κB (NF-κB) and altered expressions for inflammatory cytokines and major histocompatibility complex (MHC) molecules (7). The benefit of glucocorticoid, the only proved beneficial therapy, have been explained by their immunosuppressive properties (8). Furthermore, the immune system is also likely to be an important factor influencing the outcomes of some gene therapies and stem cell therapies for DMD since these methods can introduce exogenous protein in patients and animal models and stimulate the immunity (9, 10). Although inflammation is a hallmark of dystrophic muscular disorders, the mechanisms of its influence on muscle fiber pathology, and the involvement of factors mediating these immune mechanisms are as yet not fully understood.

The *HLA* complex is a cluster of linked genes located on the short arm of chromosome 6. Studies report that certain *HLA* types play essential roles in transplantation (11), transfusion (12), and immunogenetics (13). The *HLA* complex is highly polymorphic, and numerous diseases linked to various polymorphisms are now known. Although there has been reports describing a possible association between *HLA* alleles and DMD, these studies used low-resolution *HLA* gene typing data (14, 15). As of now, due to a lack of information with high-resolution *HLA* gene typing data, the associations between exact *HLA* alleles and DMD still remain unclear.

In this study, we use high-resolution HLA gene typing data to investigate the relationships between HLA polymorphisms and DMD. By comparing HLA allele frequencies between a group of DMD patients and a healthy control population, we also, for the first time, report associations between HLA alleles and the severity of DMD.

## Materials and methods

### Study population

Sixty-four unrelated patients from southern China diagnosed with DMD (63 males, 1 female), admitted to the Neuromuscular Clinic of The First Affiliated Hospital, Sun Yat-sen University for regular visits, and their biological mothers were enrolled in this study. A total of 503 unrelated healthy people (316 males and 187 females) living in southern China were enrolled in the study as a control group. The diagnosis of DMD for all patients was made by clinical investigation of pathological manifestations, biochemical changes, and molecular analysis; some patients also underwent a muscle biopsy for the diagnosis. Among the 64 patients in the study, the mutations in the *DMD* gene of 31 patients were found to be inherited from their mothers, while 14 patients were identified as having *de novo* mutations; the mutations found in 19 patients could not be classified due to a lack of genetic data on the *DMD* gene from their mothers. All participants were subjected to *HLA-A, -B, -C, -DRB1*, and *-DQB1* gene typing at the Department of Tissue Typing Center, Nanfang Hospital, Southern Medical University, Guangzhou, Guangdong, China.The Ethics Committee of the First Affiliated Hospital of Sun Yat-sen University approved the protocol for this study.

### Sample processing and genotyping

Venous blood (2 mL) was collected in acid citrate dextrose (ACD-B) anticoagulant tubes from each member of the 64 patients, 31 female carriers, and 503 healthy control subjects enrolled in the study. Total DNA from each blood sample was extracted by TIANamp Genomic DNA kit according to the manufacturer's instructions (Tiangen, Beijing). A Nanodrop 2000 spectrophotometer (Thermofisher scientific, USA) was used to detect the quantity and quality of the extracted DNA. On the basis of the *HLA* reference sequence of the human genome (www.Ncbi.nlm.nih.gov/genbank), amplification primers for *HLA-A, -B, -C, -DRB1, and -DQB1* for DMD patients and DMD female carriers, and *HLA-A, -B*, and *-DRB1* for the healthy controls were synthesized. PCRs were carried out using the high fidelity StrAtagene enzyme (Tianjin Super Biotechnology Developing Co., China) in a reaction volume of 50 μL also containing 2X GC buffer, 25 mM dNTP, 10 μM forward primer, 10 μM reverse primer, 2.5 U Puf enzyme, and 100 ng DNA. The reaction mix was incubated for 25 s at 96°C for an initial denaturation, followed by 5 cycles of the reaction under the following conditions: denaturation for 20 s at 96°C, hybridization for 50 s at 65°C, and elongation for 60 s at 72°C; this was followed by 20 cycles of the reaction under the following conditions: denaturation for 20 s at 96°C, hybridization for 50 s at 62°C, and elongation for 60 s at 72°C; a final elongation phase for 5 min at 72°C was also carried out. The PCR products obtained were purified by ExoSAP-IT kit (Thermo Fisher Scientific, USA) and sequenced by the Sanger method. The software Assign 3.5 SBT (Life technologies Inc, USA) was used to analyze the sequencing results.

### Evaluation of motor function

The clinical datas including age, motor functions and time period for which patients were treated with prednisone (0.3 mg/kg) before motor function assessment of 41 patients were assessed due to some of the patients did not come back for regular visit and the current clinical material were not enough for the assessment. Among all the patients, 41 patients were assessed the lower limb motor function, using Vignos scale (16). Patients were required to walk, climb stairs under protection of their parents, and rise from chairs. Patients capable of walking and climbing stairs without assistance were scored as grade 1; walking and climbing stairs with the aid of the railing were scored as grade 2; walking and climbing stairs with the aid of the railing and requiring >25 s to complete eight standard steps were scored as grade 3; walking unassisted and rising from chairs, but who were unable to climb stairs were scored as grade 4; walking unassisted, but who were unable to rise from chairs or climb stairs were scored as grade 5; walking only with assistance or walking independently with long leg braces were scored as grade 6; walking in long leg braces, but requiring assistance for balance were scored as grade 7; standing in long leg braces, but unable to walk, even with assistance, were scored as grade 8; requiring a wheelchair were scored as grade 9; and confined to bed were scored as grade 10. All 41 patients were old enough to understand and perform the instructed movements. For patients who were unable to travel to the clinic, the tests were conducted at home, and their parents were asked to provide the details of the motor activity tests.

### Statistical analysis

All statistical analyses were performed using SPSS version 20.0 (IBM Corp., Chicago, IL, USA) and GraphPad PRISM version 7.01 (GraphPad Software, San Diego, CA, USA). For all tests, a *p*-value of < 0.05 was considered statistically significant. Allele Frequency was defined as the total number of copies of the allele in the population sample (Alleles/2n). Kolmogorov–Smirnov tests were applied to all quantitative datasets to test if the data were normally distributed. The distributions of *HLA* allele frequencies between DMD patients and normal controls were compared using the Chi-square test (with Yates' continuity correction) or Fisher's exact test. Adjustments to account for multiplicity were done using Bonferroni corrections for all comparison analyses by multiplying the obtained *p*-values with the numbers of alleles detected in each *HLA* region (*HLA-A, -B*, and *-DRB1*) for each group. The association between Vignos scores of DMD patients and HLA alleles was represented by odds ratio (OR), which was estimated by multiple linear regression analysis adjusted for the age and time period (in months) for which patients were treated with prednisone before motor function assessment. Assumptions on linearity, normality, multicollinearity, and outliers were assessed and accounted for.

## Results

### Comparisons of allele frequencies of different *HLA* types between patients with DMD and the control group

**Figure 1:**
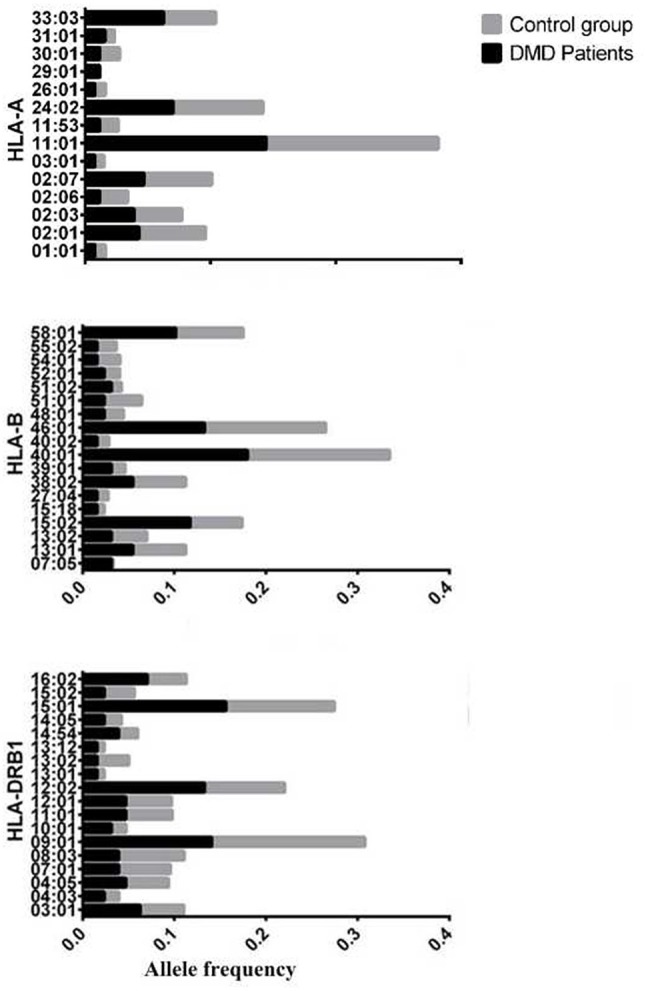
Allele frequency of HLA-A,B,DRB1 between patients with DMD and normal control, considering the allele frequencies of these alleles are>1% in either patients with DMD or control.

The allele frequency of *HLA* types in 64 patients with DMD were determined, and the number of alleles detected for each *HLA* type were 16, 29, 20, 22, and 14 for *HLA-A, -B, -C, -DRB1*, and *-DQB1*, respectively. *HLA* types with allele frequencies < 1% in both groups (DMD patients and normal control) were excluded from further analyses, which left 14, 23, and 18 alleles from *HLA-A, -B*, and *-DRB1*, respectively for further comparisons (Figure 1, Table 1). The allele frequencies of HLA-A^*^29:01, HLA-B^*^07:05, and HLA-B^*^15:02 were significantly higher in the DMD patient group than in the healthy control group while no difference of allele frequencies for *HLA-DRB1* was found (Table 1). After adjustment for multiple comparisons, the allelic frequency of HLA-B^*^07:05 was still found to be significantly higher in DMD patients than in the controls (OR = 16.19, 95%CI = 2.94–89.32, *Pc* < 0.046).

**Table 1:** Comparison of allele frequencies in *HLA-A, HLA-B, HLA-DRB1* between patients with DMD and healthy control from Southern China.

**HLA alleles^a^**	**Control (2n = 1006)**	**DMD patients (2n = 128)**	***P***	**P_C_**	**Odds ratio (95%CI)**
**HLA-A**					
01:01	17 (0.0169)	2 (0.0156)	ns	ns	0.92 (0.21–4.04)
02:01	107 (0.1064)	11 (0.0859)	ns	ns	0.79 (0.41–1.51)
02:03	77 (0.0765)	10 (0.0781)	ns	ns	1.02 (0.52–2.03)
02:06	45 (0.0447)	3 (0.0234)	ns	ns	0.51 (0.16–1.67)
02:07	109 (0.1084)	12 (0.0938)	ns	ns	0.85 (0.46–1.59)
03:01	15 (0.0149)	2 (0.0156)	ns	ns	1.05 (0.24–4.64)
11:01	276 (0.2744)	37 (0.2891)	ns	ns	1.08 (0.72–1.62)
11:53	30 (0.0298)	3 (0.0234)	ns	ns	0.78 (0.24–2.60)
24:02	144 (0.1431)	18 (0.1406)	ns	ns	0.98 (0.58–1.66)
26:01	17 (0.0169)	2 (0.0156)	ns	ns	0.92 (0.21–4.04)
29:01	1 (0.0010)	3 (0.0234)	5.17 * 10^−3^	ns	24.12 (2.49–233.65)
30:01	32 (0.0318)	3 (0.0234)	ns	ns	0.73 (0.22–2.42)
31:01	16 (0.0159)	4 (0.0313)	ns	ns	2.00 (0.66–6.07)
33:03	84 (0.0835)	16 (0.1250)	ns	ns	1.57 (0.89–2.77)
**HLA-B**					
07:02	12 (0.0119)	1 (0.0078)	ns	ns	0.65 (0.08–5.06)
07:05	2 (0.0020)	4 (0.0312)	1.94 * 10^−3^	4.46 * 10^−2^	16.19 (2.94–89.32)
13:01	58 (0.0577)	7 (0.0547)	ns	ns	0.95 (0.42–2.12)
13:02	39 (0.0388)	4 (0.0313)	ns	ns	0.80 (0.28–2.28)
15:01	36 (0.0358)	1 (0.0078)	ns	ns	0.21 (0.03–1.56)
15:02	57 (0.0567)	15 (0.1172)	8.17 * 10^−3^	ns	2.21 (1.21–4.03)
15:18	8 (0.0080)	2 (0.0156)	ns	ns	1.98 (0.42–9.43)
27:04	12 (0.0119)	2 (0.0156)	ns	ns	1.32 (0.29–5.94)
35:01	34 (0.0338)	1 (0.0078)	ns	ns	0.23 (0.03–1.66)
38:02	58 (0.0577)	7 (0.0547)	ns	ns	0.95 (0.42–2.12)
39:01	15 (0.0149)	4 (0.0313)	ns	ns	2.13 (0.70–6.52)
40:01	156 (0.1551)	23 (0.1797)	ns	ns	1.19 (0.74–1.93)
40:02	13 (0.0130)	2 (0.0156)	ns	ns	1.21 (0.27–5.44)
40:06	18 (0.0179)	1 (0.0078)	ns	ns	0.43 (0.06–3.27)
44:03	15 (0.0149)	1 (0.0078)	ns	ns	0.52 (0.07–3.97)
46:01	133 (0.1322)	17 (0.1328)	ns	ns	1.01 (0.59–1.73)
48:01	21 (0.0209)	3 (0.0234)	ns	ns	1.13 (0.33–3.83)
51:01	41 (0.0408)	3 (0.0234)	ns	ns	0.57 (0.17–1.85)
51:02	11 (0.0109)	4 (0.0313)	ns	ns	2.92 (0.92–9.30)
52:01	17 (0.0169)	3 (0.0234)	ns	ns	1.40 (0.40–4.83)
54:01	25 (0.0249)	2 (0.0156)	ns	ns	0.62 (0.15–2.66)
55:02	21 (0.0209)	2 (0.0156)	ns	ns	0.75 (0.17–3.21)
58:01	74 (0.0736)	13 (0.1016)	ns	ns	1.42 (0.77–2.65)
**HLA-DRB1**					
03:01	48 (0.0477)	8 (0.0625)	ns	ns	1.33 (0.62–2.88)
04:01	10 (0.0099)	1 (0.0078)	ns	ns	0.78 (0.10–6.18)
04:03	16 (0.0159)	3 (0.0234)	ns	ns	1.49 (0.43–5.17)
04:05	47 (0.0467)	6 (0.0469)	ns	ns	1.00 (0.42–2.40)
07:01	57 (0.0567)	5 (0.0391)	ns	ns	0.68 (0.27–1.72)
08:03	72 (0.0716)	5 (0.0391)	ns	ns	0.53 (0.21–1.33)
09:01	168 (0.1670)	18 (0.1406)	ns	ns	0.82 (0.48–1.38)
10:01	16 (0.0159)	4 (0.0313)	ns	ns	2.00 (0.66–6.07)
11:01	51 (0.0507)	6 (0.0469)	ns	ns	0.92 (0.39–2.19)
12:01	50 (0.0497)	6 (0.0469)	ns	ns	0.94 (0.40–2.24)
12:02	88 (0.0875)	17 (0.1328)	ns	ns	1.60 (0.92–2.78)
13:01	8 (0.0080)	2 (0.0156)	ns	ns	1.98 (0.42–9.43)
13:02	35 (0.0348)	2 (0.0156)	ns	ns	0.44 (0.11–1.85)
13:12	8 (0.0080)	2 (0.0156)	ns	ns	1.98 (0.42–9.43)
14:54	21 (0.0209)	5 (0.0391)	ns	ns	1.91 (0.71–5.15)
14:05	19 (0.0189)	3 (0.0234)	ns	ns	1.25 (0.36–4.27)
15:01	119 (0.1183)	20 (0.1563)	ns	ns	1.38 (0.83–2.31)
15:02	33 (0.0328)	3 (0.0234)	ns	ns	0.71 (0.21–2.34)
16:02	43 (0.0427)	9 (0.0703)	ns	ns	1.69 (0.81–3.56)

*The allele frequencies of these alleles are >1% in either patients with DMD or control. Allele Frequency: Total number of copies of the allele in the population sample (Alleles/2N) in decimal format. P_C_, correction of P-value (Bonferroni adjustment); P_C_ < 0.05 is considered as significant; ns, not significant*.

To further analyse the association between HLA alleles and DMD, we classify patients with DMD into groups of patients with deno mutation (*n* = 14) and patients with inherited mutation (*n* = 31) and compare the allele frequencies in HLA-A, HLA-B, HLA-DRB1 locus of the two groups with healthy control, respectively. A total of 8 *HLA-A*, 14 *HLA-B*, and 12 *HLA-DRB1* alleles were identified in patients with *de novo* mutations (Table 2). The allele frequencies of HLA-A^*^29:01 and HLA-B^*^07:05 were found to be significantly higher in the group of patients with *de novo* mutations than in the healthy control group (*P* < 2.09^*^10^−3^ and *P* < 1.72^*^10^−4^ for HLA-A^*^29:01 and HLA-B^*^07:05, respectively) (Table 2). After adjustment for multiple comparisons, the allele frequencies of HLA-A^*^29:01 and HLA-B^*^07:05 were still found to be significantly higher in DMD patients with *de novo* mutations than in healthy controls (OR = 77.31, 95%CI = 6.79–879.73, *Pc* < 1.67^*^10^−2^; OR = 60.24, 95%CI = 9.64–376.54, *Pc* < 2.41^*^10^−3^ for HLA-A^*^29:01 and HLA-B^*^07:05, respectively). A total of 15 *HLA-A*, 20 *HLA-B*, and 16 *HLA-DRB1* types were identified in patients with inherited mutations. No significant differences in the frequencies of any alleles in any of the *HLA* types were detected between the patients with inherited DMD mutations and healthy controls (*P* > 0.05, data not shown).

**Table 2:** Comparison of allele frequencies in HLA-A, HLA-B, HLA-DRB1 between DMD patients with spontaneous mutation and healthy control from Southern China.

**HLA alleles**	**Control (2n = 1006)**	**DMD patients with spontaneous mutation (2n = 28)**	***P***	**P_C_**	**Odds ratio (95%CI)**
**HLA-A**					
02:01	107(0.1064)	4 (0.1429)	ns	ns	0.65(0.15−2.76)
02:03	77(0.0765)	2 (0.0714)	ns	ns	0.93(0.22−0.39)
02:07	109(0.1084)	4 (0.1429)	ns	ns	1.37(0.47−4.03)
11:01	276(0.2744)	6 (0.2143)	ns	ns	0.72(0.29−1.80)
11:53	30(0.0298)	1 (0.0357)	ns	ns	1.21(0.16−9.16)
24:02	144(0.1431)	6 (0.2143)	ns	ns	1.63(0.65−4.10)
29:01	1(0.0010)	2 (0.0714)	2.09 * 10^−3^	1.67 * 10^−2^	77.31(6.79−879.73)
33:03	84(0.0835)	3 (0.1071)	ns	ns	1.32(0.39−4.45)
**HLA-B**					
07:05	2(0.0020)	3 (0.1071)	1.72 * 10^−4^	2.06 * 10^−3^	60.24(9.64−376.54)
13:01	58(0.0577)	2 (0.0714)	ns	ns	1.26(0.29−5.43)
13:02	39(0.0388)	1 (0.0357)	ns	ns	0.92(0.12−6.93)
15:02	57(0.0567)	4 (0.1429)	ns	ns	2.78(0.93−8.27)
15:25	3(0.0030)	1 (0.0357)	ns	ns	12.38(1.25−122.92)
27:04	12(0.0119)	1 (0.0357)	ns	ns	3.07(0.39−24.45)
39:01	15(0.0149)	1 (0.0357)	ns	ns	2.45(0.31−19.20)
40:01	156(0.1551)	3 (0.1071)	ns	ns	0.65(0.20−2.19)
46:01	133(0.1322)	3 (0.1071)	ns	ns	0.79(0.24−2.65)
48:01	21(0.0209)	1 (0.0357)	ns	ns	1.74(0.23−13.39)
49:01	1(0.0010)	1 (0.0357)	ns	ns	37.22(2.27−610.91)
51:01	41(0.0408)	2 (0.0714)	ns	ns	1.81(0.42−7.89)
51:02	11(0.0109)	1 (0.0357)	ns	ns	3.35(0.42−26.89)
55:02	21(0.0209)	1 (0.0357)	ns	ns	1.74(0.23−13.39)
58:01	74(0.0736)	3 (0.1071)	ns	ns	1.51(0.45−5.12)
**DRB1**					
03:01	48(0.0477)	4 (0.1429)	ns	ns	3.33(1.11−9.97)
07:01	57(0.0567)	1 (0.0357)	ns	ns	0.62(0.08−4.62)
09:01	168(0.1670)	5 (0.1786)	ns	ns	1.08(0.41−2.89)
10:01	16(0.0159)	2 (0.0714)	ns	ns	4.76(1.04−21.78)
11:01	51(0.0507)	1 (0.0357)	ns	ns	0.69(0.09−5.21)
11:06	-	1 (0.0357)	2.71 * 10^−2^	ns	0.96(0.90−1.04)
12:01	50(0.0497)	3 (0.1071)	ns	ns	2.29(0.67−7.86)
12:02	88(0.0875)	3 (0.1071)	ns	ns	1.25(0.37−4.23)
13:01	8(0.0080)	1 (0.0357)	ns	ns	4.62(0.56−38.25)
13:12	8(0.0080)	1 (0.0357)	ns	ns	4.62(0.56−38.25)
14:05	19(0.0189)	1 (0.0357)	ns	ns	1.92(0.25−14.90)
15:01	119(0.1183)	5 (0.1786)	ns	ns	1.62(0.61−4.34)

*Allele Frequency: Total number of copies of the allele in the population sample (Alleles/2N) in decimal format. “–”: the allele was not been detected. P_C_, correction of P-value (Bonferroni adjustment); P_C_ < 0.05 is considered as significant; ns, not significant*.

### Comparisons of *HLA* allele frequencies between dmd female carriers and healthy controls

The allele frequency of HLA types (*HLA-A, HLA-B, HLA-C, HLA-DRB1, and HLA-DQB1*) in the 64 mothers of patients were determined. Thirty-one biological mother of the 64 DMD patients are confirmed to be the carriers for *DMD* mutations. There were no significant differences in the allele frequencies of any *HLA* types (*HLA-A, HLA-B, and HLA-DRB1*) between female carriers of the *DMD* gene and healthy controls (Data not shown).

### Association between *HLA* types and score of motor function assessment

The 41 participants who have accessed motor function test had a minimum age of 51 months, a maximum age of 146 months, the median age was 99 months (inter-quartile range = 72.50–114 months). Patients were treated with little dosage of prednisone (0.3 mg/kg) after discussing the benefit and possible side effect with their parents. The mode of time patients accepting treatment of little dosage of prednisone before assessing motor function was 7 months (inter-quartile range = 0–27.5 months). The median score of Vignos scale obtained in our tests of the 41 participants was 3 (inter-quartile range = 2–6). Using multiple linear regression analysis adjusted for the age and time period for which patients were treated with prednisone before motor function assessment, we found that the presence of HLA-A^*^0201 allele was positively associated with lower Vignos score when compared to the absence of HLA-A^*^0201 allele (β = −2.125, *P* < 0.012, CI_95%_:−3.745, −0.504). (Detailed data is shown in Table 3). Predicted Vignos score = −2.491 + 0.073(age) −2.125 (HLA-A^*^02:01 = presence). This combination of these predictors explained nearly 51.60% (*R*^2^ = 0.5160, adjusted *R*^2^ = 0.4760) of the variance in Vignos score.

**Table 3:** Association between HLA-A^*^02:01 and Vignos score.

**Number of patients**	**Age(month)**	**Time of patients accepting treatment of little dosage of prednisone (month)**	**Vignos score**	HLA-A*0201
1	82	12	2	+
2	109	49	7	–
3	146	63	3	+
4	136	76	4	–
5	75	0	1	–
6	101	44	9	–
7	67	34	2	–
8	124	66	5	–
9	56	8	1	–
10	72	0	2	–
11	70	10	2	–
12	68	3	1	–
13	63	15	2	+
14	113	0	2	+
15	108	24	9	–
16	73	0	2	–
17	93	0	3	–
18	105	0	3	–
19	74	7	2	–
20	51	9	2	–
21	145	44	7	+
22	103	14	5	–
23	120	0	9	–
24	87	9	4	–
25	139	0	7	–
26	115	53	3	+
27	99	0	3	–
28	76	0	2	+
29	81	5	2	–
30	52	0	2	–
31	62	2	2	–
32	100	21	2	–
33	108	0	9	–
34	94	0	5	–
35	73	9	2	–
36	138	44	8	–
37	99	31	1	+
38	120	0	3	–
39	137	0	8	–
40	107	0	9	–
41	68	0	2	–

+, HLA-A^*^02:01 is positive for the patient; –, HLA-A^*^02:01 is negative for the patient.

## Discussion

Patients suffering from DMD, Becker muscular dystrophy (BMD) and other inflammatory myopathies display consistently high expression levels of class I MHCs on muscle cells, whereas, normal muscles do not express these molecules; besides this, muscle biopsies of DMD and BMD patients also indicate that in these disorders, endothelial cells express class II MHCs which are not expressed either in normal muscle or in patients with neurological disorders (17). Up-regulation of TLR7 accompanied by activation of inflammatory signaling pathways is known to elevate the expression of class I and II MHCs (18). Using low-resolution *HLA* gene typing data, two studies found that the frequency distributions of some *HLA* gene types were different between DMD patients and healthy control groups (14, 15). One such study conducted on a southern Chinese population similar to this study, reported differences in allele frequencies of *HLA-A24, -A30, -B13, -B15, -B61, -B62, -DRB104, -DRB107*, and *-DRB112* between a group comprised of DMD patients (*n* = 113) and a group of healthy controls (*n* = 406); yet another study identified the occurrence of higher frequencies of *HLA-B7* and *HLA-Aw24* in a group of children with DMD (*n* = 32) as compared to normal controls (*n* = 222) (14, 15). Previous reports indicated that MHC molecule expressed on muscle tissue and *HLA* gene may be associated with DMD though the pathogenesis is not fully understood and the role of *HLA* gene polymorphism playing in DMD is not determined since the studies are limit. In this study, we explore and demonstrate the possibility of a relationship between the pathogenesis and clinical phenotypic severity of DMD and *HLA* gene polymorphism using high-resolution *HLA* gene typing data. Our results are likely to provide additional insights into our understanding of the role played by immune-mediated mechanisms in the pathogenesis and clinical phenotypic severity of DMD.

We compared the allele frequency distributions of *HLA-A*, -*B*, and *-DRB1* types between a group of DMD patients and a large group of healthy controls. We found that the allele frequencies of HLA-A^*^29:01, HLA-B^*^07:05, and HLA-B^*^15:02 were significantly higher in DMD patients than in the healthy controls. After adjustment for multiple comparisons, the allele frequencies of HLA-B^*^07:05 were still found to be significantly higher in the DMD patients than in the healthy controls. As of now, Our study confirms the results that the HLA alleles frequency vary between patients with DMD and healthy control of the previous reports, though the identities of the *HLA* alleles associated with DMD are different. This difference could be due to several reasons. Firstly, the most important thing is that the two studies cannot be compared directly since the method of HLA genotyping is different. We use PCR-SBT to reveal a high resolution of HLA genotyping in patients and control while the study of Chen et al. (14) used polymerase chain reaction-reverse sequence specific oligonucleotide (PCR-RSSO) to test low-resolution HLA genotyping. Considering the large and continually increasing number of HLA alleles, dealing with the ambiguity of most HLA typing methods is a critical challenge. The PCR-SBT method has been considered the gold standard for high-resolution definition of *HLA* (19). This method is widely used for *HLA* genotyping with higher accuracy and reliability and is able to resolve ambiguities that could not be resolved using PCR-RSSO (20). Secondly, the sample size of different studies may be a reason for the different results observed. We intend to increase the patient sample size to confirm our results, and in future studies. What is more, ethnic background and geographic variations may be another reason for the difference between our study and previous study reported by László A and Kaiser G (1983) (15). In all, to our knowledge, our study is the first to describe the allele frequency distributions of high-resolution *HLA* gene types in DMD patients, and compare them with those in normal healthy people. Our findings confirm that there are differences in allele frequencies of *HLA-A* and *HLA-B* types between DMD patients and normal healthy people.

Further analysis of our data indicates that the HLA-A^*^29:01 and HLA-B^*^07:05 allele frequencies in DMD patients with *de novo* mutations were significantly higher than in healthy controls; however, no such significant differences were found between DMD patients with inherited mutations and healthy controls. These results lead to the interesting hypothesis that HLA-A^*^29:01 and/or HLA-B^*^07:05 may be contributing to susceptibility to sporadic DMD in the Chinese Han population in Southern China. The *DMD* locus is known to have a high spontaneous mutation rate, and one third of all sporadic cases of DMD are attributed to *de novo* mutations (21); several studies also report higher spontaneous mutation rates in DMD than those predicted in theory (22–25). Furthermore, *de novo* mutation rates in DMD may also have an ethnic component (23, 25) as demonstrated by Alcántara et al. (23), who report a higher frequency of *de novo* mutations in Mexican dystrophinopathies caused by deletion mutations (which occur at a rate of 62.2%), as well as by Sakthivel Murugan et al. (25) who report a high rate of *de novo* mutations (71%) in sporadic cases of DMD in an Indian population. As there is no effective cure for DMD, prenatal diagnosis is important in reducing the birth of affected children. As of now, advancements in genetic testing, carrier diagnosis, and prenatal diagnosis have reduced the birth rates of DMD-affected children who would have inherited the disease from their mothers. Determination of the pathogenesis of DMD caused by *de novo* mutation have a more and more important significance for the prevention of births of disease-affected children. The reasons behind the high rates of *de novo* mutation in DMD are as yet unclear, though one possible reason could be attributed to the large and complex structure of the *DMD* gene, as it contains 79 exons spanning across more than 2.4 million base pairs (26).

Our study indicates that HLA alleles are linked to an increased susceptibility to *de novo* mutations in the *DMD* gene. To the best of our knowledge, ours is the first to report detailing the possible association of *HLA* with germ line mutation. The etiology and significance of this finding remains unclear. Future large-scale studies with greater population diversity are required to confirm this finding. Germ line mutations mostly arise from replication errors during oogenesis and spermatogenesis and previous studies detailing the risk contributing to germ line mutations mainly focus on paternal inheritance especially for the factor of paternal age (27). Moreover, studies have indicated that individuals may vary in their propensity to acquire germ line mutations, notably due to mutations in DNA mismatch repair genes involved with hereditary cancer syndromes (27, 28). Another study has indicated an association between germ line risk SNPs rs2395185 at 6p22.1 (HLA class II genes) and increased APOBEC3A expression and elevated *APOBEC* mutagenesis in the lungs, indicating that some *HLA* genes may alter the risk of somatic mutagenesis through interactions with DNA mismatch repair genes (29). Although there had not been any reports to date directly indicating association of *HLA* and propensity of germ line mutagenesis, previous studies focusing on an association between *HLA* and DNA mismatch repair genes in somatic mutagenesis, and a link between variants of DNA mismatch repair genes and propensity of acquiring germ line mutagenesis may offer a possible explanation for our finding. However, further study is needed. In our study, *de novo* mutations in DMD patients may mostly accrue during oogenesis (30). We believe that HLA-B^*^0705 and HLA-A^*^29:01 may be associated with the risk of spontaneous mutation of *DMD* during oocyte development and suggest acquiring related maternal samples of patients with *de novo* mutation for subsequent investigation. To determine the etiology, experimental approaches using cells and animal models are necessary. In conclusion, we cannot determine the significance and etiology of association between spontaneous *DMD* mutations and HLA alleles that we have observed, due to the limited theories in the field in previous studies. In conclusion, we provide new insights into the understanding of *de novo* mutation of *DMD* gene, which require further study.

In addition, the association between HLA alleles and spontaneous mutation of DMD in our study may broaden understanding in prenatal diagnosis, which currently is only used in DMD carriers, while HLA alleles can be used to identify parents at a high risk of having DMD-affected children. Prenatal diagnosis of DMD should be considered in parents with positive HLA alleles, on the basis of further study that may confirm this association.

This study also investigated the existence of associations between specific HLA alleles and clinical phenotypic severity of the disease. We discovered that the HLA-A^*^02:01 allele is associated with better Vignos scores in DMD patients. This may mean that the presence of the HLA-A^*^02:01 allele could dampen the progression of DMD.

CD4+ T cells and CD8+ T cells infiltration in DMD muscle lesions have been confirmed and examined critically in humans and animal models. Several studies have indicated that the T cells involved in these inflammatory processes react specifically to dystrophin peptides presented by class I and II MHCs expressed on revertant myofibers (31, 32). Furthermore, increasing age in humans was found to correlate with an increased risk of developing anti-dystrophin T cell populations, and that eliminating either the CD4+ or CD8+ T cell populations had a beneficial effect on muscle histopathology in DMD patients (33, 34, 35). However, the role of the T cell response in the pathogenesis of DMD is still not fully understood. HLA-A^*^02:01 is associated with CD8+ T cells immunity as a MHC class I molecule. The presence of the HLA-A^*^02:01 allele has been reported to have a protective effect against multiple sclerosis in both human populations and animal models; this could be attributed to the role played by HLA-A^*^02:01 in mediating the negative thymic selection of autoreactive CD8+ T cells, which greatly reduced their numbers in the periphery (36, 37). Our data indicate that there is an association between the clinical phenotypic severity in DMD and the presence of the HLA-A^*^02:01 allele implying that MHC class I genes are likely to be involved in the pathogenesis of DMD. We hypothesize that the HLA-A^*^02:01 allele alleviates clinical phenotypic severity of DMD in a manner similar to that in multiple sclerosis, where this allele mediates CD8+ T cell responses by reducing the numbers of autoreactive CD8+ T cells in the body by influencing thymic selection. Further study with a larger number of samples and animal models needs to be carried out to investigate this hypothesis and explore the proposed mechanism.

In conclusion, this study suggests that certain *HLA* types may be associated with the pathogenesis of DMD in a southern Chinese population. Our findings may provide further insights into the importance of immune-mediated mechanisms in the pathogenesis of DMD. Future large-scale studies with more ethnicities are still needed to confirm our findings, testing HLA alleles at *HLA-A, B, C, DR*, and *DQ* loci and other relatively rare alleles like *HLA-DP*. What is more, detecting whether there is difference of HLA haplotype between patients of DMD and control may be helpful. The etiology of HLA alleles associated with *de novo* mutations and the phenotypic severity of DMD should be confirmed and studied in experimental methods using cell and animal model. Further study can also focus on identifying whether the HLA allele is involved in the pathogenesis of cardiomyopathy and neurodevelopmental disorders, which are secondary symptoms in DMD and show diverse clinical manifestations even when patients have the same *DMD* mutation.

## Ethics statement

This study was carried out in accordance with the recommendations of guidelines for clinical study, ICE for Clinical Research and Animal Trials of the First Affiliated Hospital of Sun Yat-sen University. The protocol was approved by the ICE for Clinical Research and Animal Trials of the First Affiliated Hospital of Sun Yat-sen University. This study was approved to waive the informed parental consents by ICE for Clinical Research and Animal Trials of the First Affiliated Hospital of Sun Yat-sen University, because it was impossible to get written informed parental consents from every participant, and this study did not present personal information and was not harmful to any participant.

## Author contributions

HL designed the study, analyzed the data, and drafted the manuscript. LX and ML tested the HLA alleles of participants. LW, JL, MC, RH, and YZ assisted in clinical data collection. CZ assisted in data analysis and in drafting the manuscript.

### Conflict of interest statement

The authors declare that the research was conducted in the absence of any commercial or financial relationships that could be construed as a potential conflict of interest.
